# Concentrated micro choice-based treatment for type 2 diabetes is associated with decreased risk of sleep apnoea, less daytime sleepiness and lower insomnia symptoms. A non-randomized pre – post pilot study

**DOI:** 10.3389/frsle.2025.1639626

**Published:** 2025-09-15

**Authors:** Ane Wilhelmsen-Langeland, Karsten Amund Olson, Markus Rød Kjøde, Sigurd William Hystad, Gerd Kvale, Eirik Søfteland

**Affiliations:** ^1^Division of Psychiatry, Haukeland University Hospital, Bergen, Norway; ^2^Department of Health and Caring Sciences, Western Norway University of Applied Sciences, Bergen, Norway; ^3^Department of Clinical Science, University of Bergen, Bergen, Norway; ^4^Department of Psychosocial Science, University of Bergen, Bergen, Norway; ^5^Safe Choice Clinic and Research, Bergen, Norway

**Keywords:** type 2 diabetes, sleep apnoea, insomnia, concentrated treatment, sleep

## Abstract

**Aims:**

Type 2 diabetes and sleep disorders are closely connected. We have evaluated a 4-day concentrated transdiagnostic, micro choice-based and interdisciplinary group treatment for patients with type 2 diabetes, with the aim of improving patient activation. We wanted to explore whether sleep problems would decrease after this intervention.

**Methods:**

Patients were assessed pre – post the intervention by Insomnia Severity Index (ISI), Bergen Insomnia Scale (BIS), Epworth Sleepiness Scale (ESS), Berlin Questionnaire (BQ) at baseline, 3-, 6- and 12 months follow-up, to measure symptoms related to insomnia, Obstructive Sleep Apnoea (OSA) and daytime sleepiness. Seventy-five patients with type 2 diabetes were included in our sample.

**Results:**

At baseline, 41% and 25% had symptoms of insomnia, as defined by the BIS and ISI, respectively. Another 56% were likely to suffer from OSA using the BQ. At follow-up, the intervention was associated with reduction in symptoms of sleep disorders, and the reduction was still significant at 12 months follow-up.

**Conclusions:**

Although the study was not designed to disentangle how much of the improvement was due to sleep education and medication, the outcome still shows the usefulness of the format also in terms of sleep disorders in type 2 diabetes.

## Introduction

There is an increasing recognition of the relationship between type 2 diabetes and sleep disorders. As there is a growing population of patients with type 2 diabetes (International Diabetes Federation., 2021), it is important to recognize risk-factors and develop useful treatment strategies for type 2 diabetes and related complications. The reported association between sleep disruption and type 2 diabetes is believed to be related to energy homeostasis, insulin resistance, beta-cell function (Antza et al., 2021) and autonomic neuropathy (Bhati and Hussain, 2019).

Insomnia and obstructive sleep apnoea (OSA) can negatively affect health outcomes (Tasbakan et al., 2018). A higher prevalence of insomnia in patients with OSA have led to a new term; comorbid insomnia and OSA (COMISA; Ragnoli et al., 2021). Several studies found that sleep disorders, such as obstructive sleep apnoea and insomnia, were prevalent in patients with type 2 diabetes (Koopman et al., 2020; Reutrakul and Mokhlesi, 2017; Schipper et al., 2021). A review article found the prevalence of OSA in patients with type 2 diabetes to range from 55 to 86% (Reutrakul and Mokhlesi, 2017). A meta-analysis (2020) based on studies from PubMed and Embase from countries in various parts of the world, found that the pooled prevalence of insomnia symptoms in patients with type 2 diabetes was 39%, albeit with a very low GRADE of evidence (Koopman et al., 2020). Although we know that the prevalence is high, the impression is that a focus on sleep in the general diabetes care is severely limited. A recent population-based cross-sectional study report that sleep impairment was associated with increased diabetes distress in individuals with type 2 diabetes (Riise et al., 2025). Hence, diabetes distress and sleep impairment may be interconnected.

As part of the “Project development of smarter health solutions” (PUSH-project), a concentrated transdiagnostic, micro choice-based and cross-disciplinary group treatment approach was developed to treat patients with anxiety and with depression, lower back pain, chronic obstructive pulmonary disease, long COVID and type 2 diabetes (Kvale et al., 2021). Treatment was given over 3–4 consecutive days and focused on increased awareness toward making health promoting micro-choices rather than focusing on symptoms and integrating these deliberate changes into everyday life. Patients with type 2 diabetes answered questionnaires (described below) related to sleep disorders at baseline, 3-, 6- and 12 months. Previous studies on concentrated treatment for OCD (Hagen et al., 2021) and for anxiety and for depression (Wilhelmsen-Langeland et al., 2024) have shown significant reduction in insomnia symptoms after treatment, although that was not the target of the intervention (Nordahl et al., 2018).

The micro-choice approach intervention has through qualitative interviews been described as a game-changer and that new knowledge, particularly about micro-choices and the focus on how insulin works in the body, led to change in their every-day self-management (Bendixen et al., 2025a,b). The intervention for people with type 2 diabetes, was delivered by an interdisciplinary team consisting of endo endocrinologist, psychologist, diabetes nurses, pharmacists, a nutritionist, and a physical therapist. The team collaborated closely on common goals. The intervention consisted of 3 phases, (1) a 3- to 4-week preparation phase (mostly digital), (2) a 4-day on-site group intervention, and (3) a 12-month digital follow-up. In addition to aiming at increasing the patients‘ diabetes knowledge, the cornerstones of the intervention were preparation for deliberate change, a focus on the many micro-choices one makes in everyday life, and the integration of these changes into everyday life with a minimum of follow-up from professionals. One of the focuses in the educational part of the intervention was sleep and bodily rhythms, where sleep regulation, sleep hygiene and a brief introduction of methods to alleviate insomnia symptoms were highlighted.

The aim of this study was to determine the prevalence of sleep problems and disorders in a sample of type 2 diabetes patients, and to describe possible changes in symptoms of insomnia, daytime sleepiness and symptoms related to risk of sleep apnoea from pre- to post-intervention at 3-, 6- and 12 months follow-up.

## Materials and methods

This study was part of the PUSH-project, a collaboration between Haukeland University Hospital (Bergen, Norway) and Helse i Hardanger (HiH; Øystese, Norway) which aim was to develop and test more efficient and cost-effective treatments for the growing number of chronic illnesses, and deliver it in a way that may be implemented in the public health care (Kvale et al., 2021, 2024).

For all groups, inclusion criteria were fluency in oral and written Norwegian, access to a smartphone and sufficient digital competence to handle online questionnaires. Exclusion criteria for all groups were cognitive failure, lack of self-reliance in daily routine, severe mental health problems preventing engagement in the rehabilitation program and/or conditions that prohibit physical activity. For the type 2 diabetes patient group inclusion criteria were also type 2 diabetes, age > 18 years, presence of at least one of the following complications or challenges: dysglycaemia, frequent or severe hypoglycaemia, weight gain, diabetes complications, and diet-, physical activity- and/or medical treatment challenges. Exclusion criteria for the type 2 diabetes patient group were type 1 diabetes, monogenic diabetes, secondary diabetes (pancreatogenic or any other any form of secondary diabetes), ongoing pregnancy.

### Subjects

A total of 75 patients, 39 women and 36 men, were included in the sample with a mean age of 62 years (see Table 1). Self-reported mean duration of type 2 diabetes in the patients was 13.4 (*SD* = 9.2) years. In the patient sample 45% were working full-time, 28% were on sick leave, and 27% were retired. Use of antihypertensive medication and statins, including ezetimibe, was also noted.

**Table 1:** Baseline data.

**Participants**	
Mean age (range), years	62 (20–84)
Gender (male/female)	36/39
Diabetes duration (SD), years	13.4 (9.2)
Work situation (full-time/sick leave/retired), %	45/28/27
Statin use, % of patients	64
Ezetemibe use, % of patients	12
Median number of antihypertensives (range)	1 (0–6)

Baseline data of the included participants. SD, standard deviation.

Regarding diabetes medication, some adjustments were made during the intervention (see Table 2). The table describes number of patients using different medication before and at the end of the concentrated intervention. In particular a large proportion of patients initiated GLP-1 agonists or SGLT-2 inhibitor treatment.

**Table 2:** Diabetes medications at baseline and at the end of the concentrated intervention.

**Medication group**	**Baseline *n* (%)**	**End of intervention *n* (%)**
Biguanides (metformin)	54 (75)	54 (75)
Sulphonylureas	9 (13)	2 (3)
SGLT-2 inhibitors	23 (32)	38 (53)
Thiazoledinediones	3 (4)	3 (4)
DPP-4 inhibitors	18 (25)	15 (21)
GLP-1 agonists	29 (40)	44 (61)
Long-acting insulin	29 (40)	30 (42)
Median number of units/day (range)	44 (16–100)	38 (8–100)
Short-acting insulin	10 (14)	13 (18)
Median number of units/day (range)	20 (0–100)	24 (5–90)

Data are provided as absolute number of users (n) followed by percent of users (%), or median followed by (range). SGLT-2, Sodium Glucose Transporter-2; DPP-4, Dipeptidyl Peptidase-4; GLP-1, Glucagon-Like Peptide 1.

### Training and staffing

The treatment was performed by a multidisciplinary team. The diabetes team consisted of endocrinologists, diabetes nurses, a psychologist, a pharmacist, physical therapists and a clinical nutritionist. All were trained by participating in 4-day treatment groups in a master-novice manner where they first observed a minimum of one 4-day treatment group (led by a master) before they led one. For the present study, all groups were led by the same group leaders, a psychologist (first author AWL) as main leader and a diabetes nurse as co-therapist. The group leader had observed and been co-therapist in seven 4-day treatment groups for other disorders before leading those for type 2 diabetes. Several from the interdisciplinary team joined parts of the 4-day intervention and we used manuals and step-by-step group leader instructions to ensure fidelity.

### Referral procedure

The patients were referred by their general practitioner or other physician responsible for the treatment of their type 2 diabetes. Eligible patients were invited to participate in the programme. Upon referral, the patients received a telephone call by a clinician, who informed them about the intervention and the PUSH-project. A week prior to the intervention, the patients were contacted by a therapist to ensure all necessary information about the program had been given.

### The intervention

For a more detailed explanation of the intervention, we refer to the protocol paper (Kvale et al., 2021). In short, the intervention consisted of three phases: preparing for change (phase 1), The concentrated intervention (phase 2), Integrating change into everyday life (phase 3). In general, the treatment focused on what the patient can do something about in terms of behavioral change (i.e., movement that increases insulin sensitivity) and not only on the result (i.e., weight, blood glucose, Hba1c) which is not subject to immediate change.

The concept of micro-choices entails identifying when symptoms, habits, or automated and unhelpful behavior occur (Bendixen et al., 2025b). By intentionally breaking unhelpful patterns, replace them with health-promoting micro-choices, the aim is to increase flexibility and a sense of being in control. A shift in focus was introduced from targeting glucose levels to monitoring the everyday micro-choices that may lead to increased insulin sensitivity. A shift away from trying to regulate the blood-sugar level to focus on something controllable; the actions (eating, activity, regulate stress, sleep) that impact insulin sensitivity, has the potential to increase a sense of being more in control of ones‘ illness. This shift implies that change is within reach, as opposed to physiological parameters that are not under immediate control such as blood-sugar levels or weight. The intervention also used the concept of individual goal setting. The individual goals were Specific, Measurable, Achievable, Relevant, and Time-specific according to SMART-goals (Bjerke and Renger, 2017). The sleep education lasted about 1 h and was delivered as a lecture by a somnologist (certified sleep expert) with wide clinical experience in treating insomnia and other sleep disorders. This knowledge was incorporated in other lectures as well so that all disciplines gave lectures that built on each other.

### Preparing for change

A group introduction meeting was held during this time, in which essential topics, such as challenging behavioral patterns, therapist-assisted behavior activation and basic bodily rhythms were covered. At the same meeting, the necessity of actively choose to participate in all aspects of the program was emphasized. If the patients were reluctant, they were recommended to wait until ready.

### Concentrated group intervention

A multidisciplinary approach was used during the concentrated treatment. Interactive lectures were given in groups of 6–10 patients, and were held by a diabetes nurse, a diabetes psychologist, a pharmacist, and clinical nutritionist. Lecture topics were on how to achieve behavioral change, diabetes type 2, nutrition, medication, sleep-wake rhythms, brief mindfulness-sessions, and exercise sessions. Patients were concomitantly given practical information and hands-on coaching regarding their SMART-goals. In short, patients must first specify and find a measurable and relevant objective, then identify current baseline to be able to concretise an achievable goal in an appropriate time frame. During the treatment, patients underwent individual consultations with the endocrinologist and diabetes nurses. The main goal of the consultation was to address struggles regarding their SMART-goals, and also suggest changes in medication if necessary.

### Post-intervention phase

The overall goal of the third phase was to help the patient integrate changes into everyday life. Seven to 10 days post-intervention a diabetes nurse conducted phone calls to each patient focusing on progress and how to maintain the change. At 3-months follow-up, the diabetes nurses were available through the project application for contact regarding progress and potential struggles.

### Instruments

Sleep disorders were assessed using the questionnaires Berlin Questionnaire (BQ), Bergen Insomnia Scale (BIS), Insomnia Severity Index (ISI), and Epworth Sleepiness Scale (ESS). The questionnaires were answered at a secure online application/website at baseline, 1 week after the treatment and at 3-, 6- and 12-month follow up.

### Berlin questionnaire

The BQ (Netzer et al., 1999) is a questionnaire for classifying patients in low or high risk of obstructive sleep apnoea. The questionnaire consists of 10 items, further divided into three categories. Category 1 is related to snoring, asking for frequency and severity; category 2 explores daytime sleepiness, including sleepiness while driving a motor vehicle; category 3 asks for presence of high blood pressure. The questionnaire also asks for age, sex, height, and weight. Categories 1 and 2 are positive if there are two points or more, category 3 is positive if the answer to item 10 (“Do you have high blood pressure?”) is yes or if the patient has a BMI >30 kg/m^2^. The patient is classified as high risk if two or more categories are positive, or low risk if one or less categories are positive.

### Bergen insomnia scale

The BIS is a self-report form that consist of six questions corresponding to the DSMV-IV criteria for insomnia (Pallesen et al., 2008). Each question ranges from 0 to 7 days per week, adding to a possible composite score ranging from 0 to 42. The first three questions concern sleep onset, maintaining sleep and preterm awakening, whilst the latter three pertain to the feeling of being rested, functioning during daytime, and being dissatisfied with current sleep. The BIS also has a dichotomous score relating to clinical likelihood of having insomnia disorder (yes or no) that corresponds to scoring 3 or higher on one of questions 1–4 and 3 or higher on one of questions 5–6.

### Insomnia severity index

The ISI is a self-report tool to measure subjective insomnia symptoms (Morin et al., 2011). The form has seven items, pertaining to severity of problems with sleep latency, early awakening, satisfaction with sleep pattern, and impact on daily functioning. Each item is scored between 0 and 4, with the total score ranging between 0 and 28. A score between 0 and 7 signifies no clinically significant insomnia, 8–14 signifies subthreshold insomnia, 15–21 and 22–28 suggest moderate and severe insomnia, respectively.

### Epworth sleepiness scale

The ESS was initially made to offer an affordable and less time-consuming way to screen for daytime sleepiness than for instance Multiple Sleep Latency Test (MSLT; Johns, 1991). It gives 8 scenarios for which the patient is to rate the likelihood of dozing off or falling asleep. The 8 scenarios are scored from 0 (“No chance of dozing“) to 3 (“High chance of dozing”), with total scores ranging between 0 and 24. A cut-off of ≥11 is considered sleepiness above normal (Johns, 1991).

### COMISA

COMISA can be calculated by high risk of OSA on the BQ in addition to “moderate” or “severe” on ISI and by high risk of OSA on the BQ in addition to fulfilling the dichotomous score relating to clinical likelihood of having insomnia disorder (yes or no) on the BIS.

### Statistical analysis

Since this is a non-randomized pre-post pilot study, no sample size calculation was made in advance. Mixed-effects regression models were used to test for pre- to post changes in continuous BIS, ISI, and ESS scores at different timepoints [1 week (BIS only), 3 months, 6 months and 12 months].

Changes in proportions scoring below or above the predefined cut-offs on ISI, BIS, BQ, and ESS were examined first with Cochran's *Q* tests. Used longitudinally as here, the Cochran's *Q* test can indicate whether the proportions in the different cut-off categories at the different time points are the same in the population. Statistically significant Cochran's *Q* tests were followed by *post hoc* McNemar's tests comparing baseline proportions to all subsequent time points. The four ISI categories were combined into two categories for the sake of these analyses: a non-insomnia/subthreshold category consisting of the first two categories and a moderate/severe insomnia category consisting of the last two categories.

There were some missing values in ISI and BQ. For ISI the missing values were *n* = 8 at 3 months follow-up, *n* = 12 at 6 months follow-up, and *n* = 30 at 12 months follow-up. For BQ missing values were *n* = 8 at 3 months follow-up, *n* = 11 at 6 months follow-up, and *n* = 23 at 12 months follow-up. Mixed-effect models are flexible and can be fitted even if some outcome data are missing and can produce unbiased estimates provided that missingness is not Missing Not at Random (MNAR). For the analyses on the continuous outcomes all participants were thus included, irrespective of missing data at any of the assessment points. We know that many participants needed help to log on to the website where the questionnaires were filled out and this fact suggests that their ability or willingness to access the site is related to their age or technical skills, which are likely associated with underlying factors such as digital literacy. This indicates that the missing data are systematically related to unobserved characteristics (e.g., comfort with technology), meaning that the data are MNAR but are instead missing due to these underlying factors.

To address missing data on the categorical outcomes, multiple imputation was performed using chained equations (MICE). A combination of logistic and ordinal regression models was used to generate 30 datasets. Following imputation, analyses were conducted separately within each dataset. As Cochran's Q and McNemar's χ^2^ are not parameter estimates, it does not make sense to use Rubin's rule to pool them across imputations. Instead, we used the range of *Q* and χ^2^ statistics and associated *p*-values across imputations to assess the consistency and robustness of the findings. Although the original sample size was 75, the number of complete cases available for analysis after imputation varied slightly across time points, due to residual missingness in variables not fully resolved by the imputation models. For descriptive purposes, the average proportion of participants classified as high risk on ISI and BQ were calculated across the 30 datasets and are presented in the Results section.

### Ethics

Written consent was signed digitally by all participants before data collection. The project was approved by the ethics committee in Western Norway (REK West 2020/101.648) and was conducted in accordance with the Helsinki declaration. ClinicalTrials.gov Identifier: NCT05234281.

## Results

### Epworth sleepiness scale

The mixed-effects regression showed that total ESS score decreased from baseline to 1-week post-treatment. At 3-month follow-up reduction in score from baseline was −1.56 [*b* = −1.56, (95% CI: −2.78, −0.34), *p* = 0.013], at 6-months −2.39 [*b* = −2.39, (95% CI: −3,61, −1,16), *p* < 0.001], and at 12-months −4.31 [*b* = −4.31, (95% CI: −5.53, −3.08), *p* < 0.001]. Table 3 presents additional marginal means computed from the mixed regression.

**Table 3:** Estimated marginal means (SE) from mixed linear regression and pairwise comparisons between pre- and post-measures.

**Instruments**	**Pre**	**1-week**	***p***	**3-months**	***p***	**6-months**	***p***	**12-months**	***p***
ESS	8.35 (0.61)			6.79 (0.61)	=0.013	5.96 (0.61)	< 0.001	4.04 (0.61)	< 0.001
ISI	9.45 (0.69)			7.86 (0.71)	< 0.01	7.65 (0.72)	< 0.01	6.98 (0.77)	< 0.001
BIS	14.96 (1.15)	13.10 (1.16)	=0.046	14.85 (1.17)	=0.914	11.43 (1.31)	< 0.01	11.94 (1.29)	< 0.01

ESS, Epworth sleepiness scale (range 0–24); ISI, Insomnia severity index (range 0–28); BIS, Bergen insomnia scale (range 0–42). All statistical comparisons are between the pre-measures and the subsequent follow-up measures.

At baseline, 28% of patients (*n* = 21) scored above the cut-off on ESS (see Table 4). The proportion decreased to 21.3% at 3-month follow-up, 18.7% at 6-months, and 12% at 12 months. The Cochran's *Q* was statistically significant, *Q*(3) = 10.571, *p* = 0.014, and the *post hoc* McNemar's tests showed that the proportion scoring 11 or above was statistically significantly lower at 12-month follow-up than at baseline, χ^2^(1) = 8, *p* < 0.01. There were no statistically significant differences between baseline and either 3-month or 6-month follow-up.

**Table 4:** Percentage of patients categorized as at risk at the different time points.

**Instruments**	**Baseline**	**1-week**	**3-months**	**6-months**	**12-months**
ESS % (>11)	28.0 *(n = 21)*	—	21.3 *(n = 16)*	18.7 *(n = 14)*	**12.0** ***(n** **=** **9)***
ISI %^a^ (moderate/severe)	25.3 (*n = 19*)	—	**9.0** ***(n** **=** **6)***	**11.1** ***(n** **=** **7)***	**6.7** ***(n** **=** **3)***
BIS % (likely insomnia)	58.7 *(n = 44)*	50.7 (*n* = 38)	61.3 *(n = 46)*	**25.3** ***(n** **=** **19)***	**29.3** ***(n** **=** **22)***
BQ %^a^ (High-risk)	56.0 *(n = 42)*	—	**38.8** ***(n** **=** **26)***	**28.1** ***(n** **=** **18)***	**28.9** ***(n** **=** **15)***

ESS, Epworth sleepiness scale, the proportion of which scored above 11 indicating sleepiness above normal at each time-point. ISI, Insomnia severity index, the proportion indicates moderate and severe scores on the insomnia index. BIS, Bergen Insomnia Scale, the proportion indicates likely insomnia. BQ, Berlin Questionnaire, the percentage of which were found to have a high risk of OSA.

Follow-up proportions in bold are statistically significantly different from baseline.

^a^Percentages shown are valid percentages based on complete cases.

### Bergen insomnia scale

The mixed-effects regression showed a reduction in the total BIS score from baseline to 1-week post-treatment [*b* = −1.85, (95% CI: −3.67, −0.035), *p* = 0.046, Table 3]. As can be seen in Table 3, the mean level of BIS reverted to baseline level at 3-months follow-up (*p* = 0.914), and then decreased again at 6-months [*b* = −3.52, (95% CI: −5.71, −1.34), *p* = 0.002] and 12-months follow-up [*b* = −3.01, (95% CI: −5.15, −0.87), *p* = 0.006].

At baseline, 44 (58.7%) patients out of a total 75 were categorized as likely to have insomnia (see Table 4). The Cochrane's *Q* test showed that the proportion changed over time, *Q(*4) = 45.565, *p* < 0.001. Follow-up McNemar's tests showed a statistically significant reduction in proportions at 6-months [25.3%, χ^2^(1) = 21.55, *p* < 0.001] and 12-months [29.3%, χ^2^(1) = 15.12, *p* < 0.001]. The differences in proportions were not found to be statistically significant at 1 week and 3 months follow-up.

### Insomnia severity index

The mixed-effects regression showed that total ISI scores improved from baseline to post-treatment (see Table 3). The reduction from baseline to 3-month follow-up was −1.59 [*b* = −1.59, (95% CI: −2.63, −0.55), *p* = 0.003], from baseline to 6-month follow-up −1.80 [*b* = −1.80, (95% CI: −2.86, −0.74), *p* = 0.001], and to 12-months −2.48 [*b* = 2.48, (95% CI: −3.67, −1.29), *p* < 0.001].

At baseline, 25.3% of patients fell into the moderate/severe insomnia category (*n* = 15 in moderate and *n* = 4 in severe, see Table 4). The Cochran's *Q* statistic was statistically significant in all 30 imputations, ranging between 21.091 and 27.462 (all *p*s < 0.001). Follow-up McNemar's tests showed a statistically significant reduction in proportions at 3-months (χ^2^ range: 9.31–13, all *p*s < 0.01), 6-months (χ^2^ range: 8.33–12, all *p*s < 0.01), and 12-months (χ^2^ range: 10.89–14.22, all *p*s < 0.01). The average proportion of participants classified as moderate/severe insomnia across the 30 imputations was 8.4% at 3-months (based on *n* = 73), 9.9% at 6-months (based on *n* = 72), and 4.2% at 12-months (based on *n* = 72).

### Berlin questionnaire

At baseline, 56% (*n* = 42) of patients were found to have high risk of OSA (see Table 4). The Cochran's *Q* statistic was statistically significant in all 30 imputations, ranging between 21.344 and 33.308 (all *p*s < 0.001). *Post hoc* McNemar's tests showed that the proportions having high risk of OSA were statistically significantly lower at 3-months (χ^2^ range: 5–9.78, *p* range: 0.002–0.025), 6-months (χ^2^ range: 13.37–19.59, all *p*s < 0.001), and 12-months follow-up (χ^2^ range: 6.26–18.62, *p* range: 0.000–0.012) compared with baseline. The average proportion of participants classified as high risk across the 30 imputations was 39.2% at 3-months (based on *n* = 73), 27.2% at 6-months (based on *n* = 73), and 31.9% at 12-months (based on *n* = 73).

### COMISA

At baseline, 21.3% and 36% fulfilled the definition of COMISA based on BQ + ISI and BQ + BIS, respectively. The percentages were reduced to 5.6% and 28.9% at 3 months, 4.3% and 9.6% at 6 months and 2.8% and 11% at 12-months follow up.

## Discussion

To the extent of our knowledge, this is the first study to highlight insomnia, excessive daytime sleepiness and OSA-symptoms in patients with type 2 diabetes before and after a multidisciplinary and multi-faceted group-based treatment. Although there is a substantial literature on the concomitant occurrence of sleep disorders and type 2 diabetes, little is known about the effect of a concentrated treatment addressing such issues, nor other group-based treatments. Although not a primary treatment target, we observed significant reductions in insomnia-symptoms and daytime sleepiness long-term, and reduction in the risk of OSA on all assessment points from pre to post intervention.

The validity of using self-report forms to assess for different sleep disorders, without objective measures, is limited to ascertain patients' own experiences of sleep quality, lengths, and effects on daily life. However, as the diagnosis of, for instance, insomnia is subjective and assessed by the patient and his/her physician, it is valid information to use patient self-assessment. In addition, self-report forms such as ISI have showed significant correlations with clinician-assessment, sleep diary and polysomnographic results, although polysomnographic correlation was small (Bastien et al., 2001).

By contrast to the concordance between different sleep-forms such as ESS and BQ; (Pallesen et al. 2008) found that scoring high on BIS may be negatively correlated to total scores on ESS. This has been postulated to be caused by the tendency for patients with insomnia to be hyperaroused (Riemann et al., 2010). This implies the importance of using several forms to assess for different sleep disorders. However, using too numerous forms may cause survey fatigue (O'Reilly-Shah, 2017).

Previous evidence points toward that treating the underlying chronic disease with the 4-day concentrated treatment format, also has positive effect on comorbid symptoms such as insomnia symptoms, however, the reduction in insomnia symptoms seemed to be dependent on reduction in symptoms of depression and did not occur until 3-month follow up in a sample of patients with OCD (Hagen et al., 2021). In patients with chronic depression and/or chronic anxiety however, we did observe a rapid reduction in insomnia symptoms in after just 1 week after the concentrated treatment (Wilhelmsen-Langeland et al., 2024).

Our results show that at baseline, 41.3% and 25.3% of patients had symptoms indicative of insomnia in terms of BIS and ISI (moderate to severe). Using the Berlin Questionnaire we also found at baseline that 56% of patients were likely to suffer from OSA. A high proportion of our study population fulfilled our definition of COMISA. In line with this, we found that after our treatment, patients reported improvements both on symptoms of OSA and insomnia, suggesting that our treatment benefit such patients. The reduction in insomnia symptoms is in line with previous research showing positive effects of brief behavioral treatment for insomnia (BBTI; Kwon et al., 2022). However, even BBTI is a 4-session treatment (two in-person and two telephone calls), hence our intervention of one session was more concentrated. In fact, the percentage that fulfilled the definition of COMISA was reduced by 17 and 20 percentage points at 12-months follow-up. Considering the design of the study and the lack of physiological assessment, the results do however need to be interpreted with caution.

The link between micro-choices, diabetes type 2 self-management and changes in symptoms of sleep disturbance is likely intertwined and bidirectional. The link between sleep and diabetes is believed to be related to pathophysiological mechanisms such as energy homeostasis, insulin sensitivity, beta-cell function (Antza et al., 2021) and autonomic neuropathy (Bhati and Hussain, 2019). We can merely speculate based on our design. However, increased knowledge about diabetes (heath literacy), change in medication, increased focus on exercise and food choices that help the body becoming more insulin sensitive through a myriad of healthy micro-choices and increased awareness of micro-choices relating to sleep-wake rhythms (take a 15-min walk after dinner instead of a nap) may all contribute to reduced sleep disturbance.

### Strengths and limitations

Our study design poses as a limiting factor, as the non-randomized approach might give rise to selection bias. Missing data, particularly regarding the Berlin Questionnaire, might contribute to selection bias as we cannot evaluate the condition of those not able to adhere to the follow-up forms (Lachin, 2016). We have adjusted for missing data by mixed-effect models that are flexible and can be fitted even if some outcome data are missing and can produce unbiased estimates provided that missingness is not MNAR, which we argue that is the case. We used MICE to address missing data on the categorical outcomes, where multiple imputation was performed. This procedure produces less biased parameter estimates compared to simpler methods.

As part of the intervention, changes were made in the patients' diabetes medication (see Table 2). The contributing effect of such changes on sleep problems in patients with type-2 diabetes may also be interesting to explore in future studies, as SGLT-2 inhibitors have yielded promising results for the treatment of OSA (Tanriover et al., 2023), and we cannot rule out that effect. In our sample 23% of the participants were on SGLT-2 inhibitors pre intervention while 53% were on them at the end of the intervention. Consequently, such changes might have reduced OSA symptoms in our study population. Clearly, the study design does not allow for teasing out the individual effect components and changes in medication may have had a large impact. Also, we cannot rule out drug side-effects in relation to the changes in the sleep parameters. However, we did not make changes in medication other than related to their diabetes during the intervention.

The same two group leaders led all intervention groups. Hence, we can not rule out that unique characteristics or biases of the group leaders could influence outcomes, making it difficult to determine if observed effects are due to the intervention or the leaders' individual traits.

A strength of our study is that we have investigated common sleep-related disturbances in type 2 diabetes and measured them at multiple and long-term time points. Such study designs and data are rare in type 2 diabetes, and hence provide novel and potentially important information. Also, including lectures on how to improve sleep in diabetes treatment programs is not common. The sleep-related education session lasted only 45–60 min and we do not know how much of the improvements has to with what they learned about sleep-wake rhythms (we did not test their sleep knowledge pre – post) and how much of the improvement is residual to the changes they made in activity, food intake and so on. The dismantling of these effects should be investigated in future studies. However, in previous studies from concentrated treatment format for OCD, the authors conclude that 4 days may be too short to reduce insomnia symptoms short-term (Hagen et al., 2021), while we found an immediate reduction (after 1 week) in insomnia-symptoms when including more sleep-specific education in concentrated treatment for depression and for anxiety (Wilhelmsen-Langeland et al., 2024).

If this concentrated treatment reduces the risk of OSA in patients with type 2 diabetes, it may be highly health-economically positive as other treatment modalities for OSA are costly, and so is the assessment of OSA. The combination of OSA and type 2 diabetes also increase the risk of cardiovascular disease, hence the results we report on here may be of great importance, pointing at novel ways to reduce diabetes type 2 complications in a short amount of time. A physiological assessment of OSA is however still the gold standard.

### Conclusion

The PUSH-project's goal, to explore treatment alternatives for the next generation of public health care solutions, constitutes the core value of our study. Our results indicate an effect of the 4-day concentrated treatment format on different modalities of sleep in our sample of diabetes patients. The primary goal of the treatment given was to change focus from end-results (e.g., glucose levels) to behavioral daily health-promoting micro-choices, thus emphasizing a holistic and hands-on approach. As such, the exact cause of improvement is not enlightened through our study. As sleep is a cornerstone in maintaining good health and wellbeing, it may be beneficial to look for treatment options which include sleep as an important outcome target.

## Data Availability

The datasets presented in this article are not readily available because raw data may be shared upon reasonable request, please contact the corresponding author. Requests to access the datasets should be directed to anewil@ihelse.net.
